# Open versus laparoscopic ventriculoperitoneal shunt placement in children: a systematic review and meta-analysis

**DOI:** 10.1007/s00381-023-05966-5

**Published:** 2023-05-25

**Authors:** Ladina Greuter, Linus Ruf, Raphael Guzman, Jehuda Soleman

**Affiliations:** 1 Department of Neurosurgery, University Hospital of Basel, Spitalstrasse 21, 4031 Basel, Switzerland; 2 Department of Pediatric Neurosurgery, University Children Hospital of Basel, Basel, Switzerland; 3 Faculty of Medicine, University of Basel, Basel, Switzerland

**Keywords:** Ventriculoperitoneal shunt surgery, Pediatric shunt, Laparoscopic shunt surgery, Systematic review, Pediatric neurosurgery

## Abstract

**Background:**

Ventriculoperitoneal shunt (VPS) surgery is the traditional method for treating hydrocephalus, remaining one of the most regularly used procedures in pediatric neurosurgery. The reported revision rate of VPS can reach up to 80% and significantly reduces the quality of life in the affected children and has a high socioeconomic burden. Traditionally, distal VPS placement has been achieved open via a small laparotomy. However, in adults several studies have shown a lower rate of distal dysfunction using laparoscopic insertion. As the data in children are scarce, the aim of this systematic review and meta-analysis was to compare open and laparoscopic VPS placement in children regarding complications.

**Methods:**

PubMed and Embase databases were searched using a systematic search strategy to identify studies comparing open and laparoscopic VPS placement up to July 2022. Two independent researchers assessed the studies for inclusion and quality. Primary outcome measure was distal revision rate. A fixed effects model was used if low heterogeneity (I^2^ < 50%) was present, otherwise a random effects model was applied.

**Results:**

Out of 115 screened studies we included 8 studies in our qualitative assessment and three of them in our quantitative meta-analysis. All studies were retrospective cohort studies with 590 analyzed children, of which 231 children (39.2%) received laparoscopic, and 359 children (60.8%) open shunt placement. Similar distal revision rates were observed between the laparoscopic and open group (3.75% vs. 4.3%, RR 1.16, [ 95% CI 0.48 to 2.79], I^2^ = 50%, z = 0.32, p = 0.74). There was no significant difference in postoperative infection rate between the two groups (laparoscopic 5.6% vs. open 7.5%, RR 0.99, (95% CI [0.53 to 1.85]), I^2^=0%, z = -0.03, p= 0.97). The meta-analysis showed a significantly shorter surgery time in the laparoscopic group (49.22 (±21.46) vs. 64.13 (±8.99) minutes, SMD-3.6, [95% CI -6.9 to -0.28], I^2^=99%m z= -2.12, p= 0.03) compared to open distal VPS placement.

**Conclusion:**

Few studies are available comparing open and laparoscopic shunt placement in children. Our meta-analysis showed no difference in distal revision rate between laparoscopic and open shunt insertion; however, laparoscopic placement was associated with a significantly shorter surgery time. Further prospective trials are needed to assess possible superiority of one of the techniques.

## Introduction

Ventriculoperitoneal shunt (VPS) surgery is the traditional procedure for treating hydrocephalus and remains one of the most frequently used procedures in pediatric neurosurgery [1]. Since its introduction, VPS surgery has undergone several technical improvements, however, complication rates remain relatively high [2]. The lifetime risk for a revision surgery of a pediatric VPS can reach up to 80% and significantly reduces the quality of life in the affected children [3–8]. The most common reason for revision surgery are proximal occlusions, however, distal shunt failure, either due to misplacement or obstruction are still frequently observed [7, 9, 10]. Mostly, the distal catheter is placed in the peritoneal cavity through an open mini-laparotomy, while, in adults, it was shown that laparoscopic catheter insertion significantly reduces the rate of shunt failure [11, 12]. In children, the literature is scarce and only few comparative studies exist [13–15].

The aim of this systematic review and meta-analysis was to compare the clinical outcome of open and laparoscopic VPS surgery in children.

## Methods

**Fig. 1:**
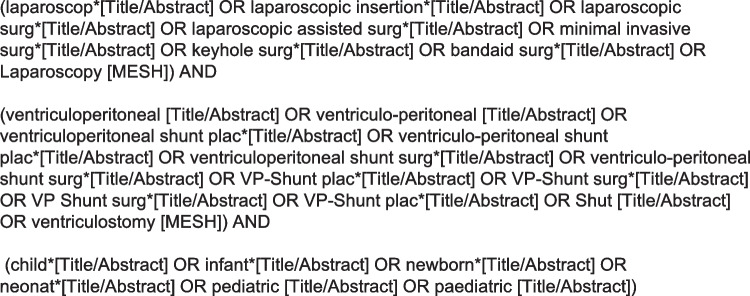
Search string used for our systematic literature review in PubMed

We followed the Preferred Reporting Items for Systematic Reviews and Meta-Analyses (PRISMA) guidelines for this systematic review [16]. The search string used for the present systematic review contained a combination of the keywords “VPS surgery”, “Children” or “Pediatric” and “Laparoscopic” (Fig. 1). The databases PubMED and Embase were searched for this systematic review and meta-analysis and all results published until 1^st^ of July 2022 were assessed by two of the authors independently (LR and LG). After removal of any duplicates, all remaining articles were screened according to their titles. The selected articles were further analysed according to their abstracts, while the remaining results underwent a full text review, and a final list of references was compiled. In case of disagreement concerning the inclusion of an article, the decision was made by a third researcher (JS).

### Inclusion criteria and outcome analysis

We included all randomized controlled trials (RCT), prospective and retrospective cohort studies, as well as case series with >5 patients comparing open and laparoscopic VPS surgery in children for the quantitative analysis. Studies only describing laparoscopic insertion were included for our qualitative review but not for the meta-analysis. Studies only focusing on laparoscopic shunt revisions and not primary insertion were excluded, as well as studies describing any other technique of VPS placement such as blind trocar insertion or similar methods, since not enough studies looking at these techniques are available, and we did not want to pool other, insertion methods together with the laparoscopic group to avoid any bias. We only included studies in English.

The primary outcome measure was distal shunt failure and subsequent revision surgery. Additional outcome measures were overall revision rate, periprocedural bowel injury, infection rate, as well as operative time in minutes.

### Quality assessment

Quality assessment of non-randomized retrospective cohort studies was carried out using the Newcastle Ottawa Scale [17]. Only studies included in the quantitative analysis were assessed.

### Statistical analysis

Pooled outcomes were either calculated with the fixed-effects model in case of low-heterogeneity (I^2^ ≤50%) between the studies or random-effects model if I^2^ was > 50%. Risk ratio (RR) was used as an effect measure for the pooled outcomes.

To better estimate a pooled incidence rate of distal revisions, overall revisions, and infections, we calculated a unilateral pooled outcome rated using a random-effects model for all studies describing laparoscopic VPS placement in children even if there was no direct comparison to open techniques described.

Forest plots were generated for all outcomes where a comparison between the studies was possible.

The analyses were carried out using R as a statistical software (R Foundation for Statistical Computing, Vienna, Austria, Version 4.0.3) running the dmetar package [18].

## Results

**Fig. 2:**
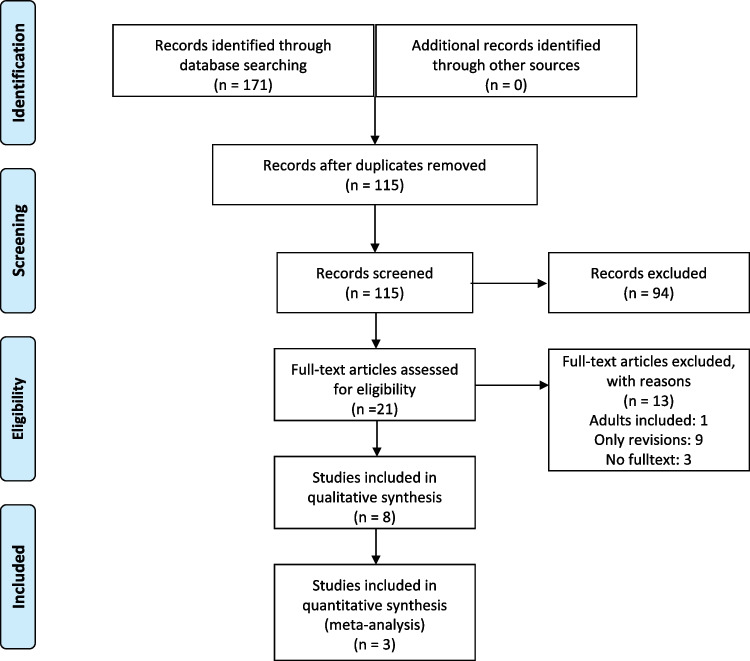
Flowchart showing the selection process of the included studies

After screening 115 articles according to their titles, a list of 21 references was compiled which underwent full-text review. Three studies were included in our quantitative analysis and five additional studies for our qualitative review (Fig. 2, Table 1) [13–15, 19–24].

**Table 1:** Overview of the included studies, NOS = Newcastle-Ottawa scale

Author and Year	Study Type	Number of laparoscopic surgeries	Number of open surgeries	NOS
Mulvaney et al. 2022	Retrospective Cohort	98	90	8
Almetaher et al. 2020	Retrospective Cohort	36		
Hanna et al. 2019	Retrospective Cohort	92	100	8
Haye et al. 2019	Retrospective Cohort	110		
Fahy et al. 2018	Retrospective Cohort	41	169	8
Soleman et al. 2016	Retrospective Cohort	20		
Handler et al. 2008	Retrospective Cohort	126		
Bani and Hassler 2006	Retrospective Cohort	39		

 A total of 562 children received laparoscopic shunt placement in the papers included in the qualitative analysis. All included studies were retrospective cohort studies or larger case series, while no randomized study comparing open and laparoscopic VPS surgery in children was published (Table 1). For the comparative quantitative meta-analysis, 231 children (39.8%), with a mean age of 4.51 (±3.13) years, received laparoscopic and 359 children (60.2%), with a mean age of 3.40 (±1.56) years, received open VPS placement [13–15].

Average follow-up time varied among the different studies ranging from 30 days to 33 months [14, 22].

### Distal revision rate

Distal revision rate was described by five of the eight studies in the qualitative review. It ranges from 0-10% for laparoscopy while for open surgery a distal revision rate of 4-5% was described [13, 14, 21, 22, 24, 25].

**Fig. 3:**
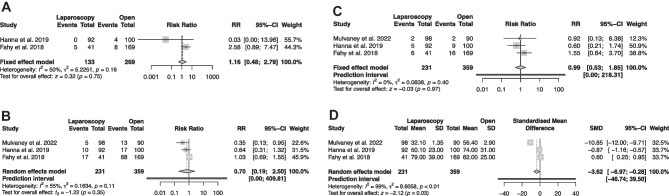
**A**) Forest plot of distal revision rate **B**) overall revision rate **C**) infection rate and **D**) duration of surgery

Quantitative analysis showed a similar overall pooled distal revision rate in the laparoscopic and open shunt group (3.75% vs. 4.3%, RR 1.16, (95% CI [0.48 to 2.79]), I^2^ = 50%, z = 0.32, p = 0.74, Figure 3A) [13, 14].

The type of distal failures varied among the different studies, while one study did not report any distal failures, two did not further specify the types [14, 22, 24]. The most common distal complications were catheter misplacement (n=6, including open placement) or migration (n=6) followed by catheter obstruction (n=5), intraabdominal malabsorption (n=5) and disconnection (n=1) [13, 21, 25]. In the retrospective cohort study by Handler et al., five cases (38.4% of all their distal complications) were caused by a broken catheter due to the split trocar used for distal catheter insertion [21]. Fahy et al. mentioned that several patients diagnosed with a proximal obstruction underwent prophylactic distal revision in the same surgery. These revisions were mainly laparoscopic and happened within the first 6 months of insertion [14].

### Overall revision rate

Overall revision rate was assessed by six studies [13–15, 21, 22, 24]. In the laparoscopic group the revision rate ranged from 2.7-41.5%, while in the open group the rate was 14.4-40.3%. Overall pooled outcome for overall revision rate in the quantitative analysis was lower in the laparoscopic group, however no statistically significant difference was seen (13.8% vs. 27.2%, RR 0.70, (95% CI [0.19-2.5]), I^2^ = 55%, t = -1.22, p = 0.35, Fig. 3B).

## Periprocedural complication rate and extent of laparoscopic surgery

Periprocedural bowel injury was only reported by one comparative study and no incident was reported in both groups [14]. None of the studies reported on bladder perforations or other intraoperative complications. In the study by Fahy et al. two patients (4%) underwent a concurrent adhesiolysis and one underwent an umbilical hernia repair (2%), while Handler described seven cases (5.2%) undergoing either adhesiolysis or catheter retrieval [14, 21]. Bani and Hassler reported two (5.1%) distal catheter removals during laparoscopy, while in the other studies, no additional abdominal surgeries were not performed or not reported [19].

Six studies used a peel-away trocar to insert the distal shunt and the placement of the peel-away sheath was visualized with the camera [13, 14, 19, 21, 22, 24]. Only one study described a three-trocar technique where the shunt was grabbed with forceps along the catheter and placed in the peritoneal cavity [23].

### Infection rate

All studies included in the qualitative review reported an infection rate. For the laparoscopic group the rate ranged from 2.1-14.6%, while for the open group it ranged from 2.2-9.5% [13–15, 21–24]. Quantitative, comparative analysis revealed a lower infection rate in the laparoscopic group, while no significant difference between the groups was observed (5.6% vs. 7.5%, RR 0.99, (95% CI [0.53 to 1.85]), I^2^=0%, z = -0.03, p= 0.97, Fig. 3C).

## Duration of surgery

All included studies reported the duration of surgery ranging from 15.4-79 minutes in the laparoscopic group and 56.4-75 minutes in the open group [13–15, 19, 21–24]. Pooled mean duration for laparoscopic surgery was 49.22 (±21.46) minutes, and 64.13 (±8.99) minutes for open VPS placement. Pooled comparative outcome analysis showed a significant shorter duration in the laparoscopic group (SMD -3.6, (95% CI [ -6.9 to -0.28]), I^2^=99%, , z= -2.12, p= 0.03, Fig. 3D).

Two studies provided more information about the duration of the procedure. Bani and Hassler reported their laparoscopic part to last 5 to 20 min, with a mean of 8 min, including two catheter removals [19].

Almetaher et al. reported a surgical duration of 126 ± 9 min in abdominal pseudocysts, 48± 5 min in recurrent inguinal hernias, 112 ± 7 min in adhesive intestinal obstruction, 37 min in a subcutaneous cyst, 25 ± 2 min for the extraction of a distal shunt tube, and 35 min for the repair of an umbilical fistula [23].

### Pooled incidence rates

**Fig. 4:**
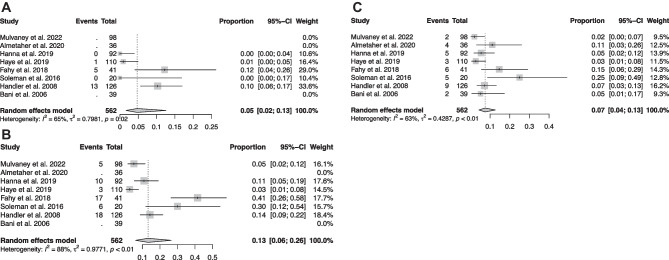
Forest plot showing pooled incidence rates of **A**) distal revisions **B**) overall revisions and **C**) infections

Pooled distal revision rate of laparoscopic VPS insertion was 5.0%, (95% CI[0.02 to 0.13], Fig. 4A) [13, 14, 21, 22, 24].

Laparoscopic VPS placement had a pooled overall revision rate of 13.8% (95% CI [0.06 to 0.26]), Fig. 4B [13–15, 21, 22, 24].

Pooled infection rate of laparoscopic VPS placement was 7.5%, (95% CI [0.04 to 0.13]), Fig. 4C [13–15, 19, 21–24].

### Quality assessment

The Newcastle-Ottawa Scale (NOS) rating of all three studies included in this meta-analysis was 8 (Table 1) [13–15].

## Discussion

This meta-analysis, comparing the distal revision rate between open and laparoscopic surgery for distal VPS placement in children, showed similar distal failure rates between the two groups, with a significantly shorter surgery time for the laparoscopic group.

### Revision & infection rate

Despite a high rate of hydrocephalus requiring shunt placement in children, there is a paucity of literature comparing open and laparoscopic shunt placement [1, 13–15]. In children, over 80% of patients undergo VPS revision surgery during a 15-year follow-up with a distal shunt revision rate of up to 15% [26]. In in this pooled outcome analysis no difference for distal or overall revision rates between laparoscopic and open VPS insertion was observed [13–15]. However, in the adult population several studies exist showing a significantly lower distal failure rate after laparoscopic distal insertion of VPS [11, 12, 27, 28]. This observed difference between pediatric and adult patients could be due to the naturally different habitus and size between adults and children. Open VPS placement in obese patients, showed a significantly higher distal failure rate and especially for such cases laparoscopic placement could have an advantage, however, obesity is much rarer in the pediatric population. In this systematic review and meta-analysis, only Fahy et al. assessed the patient cohort for obesity, which was zero in both groups, while the other studies did not report it [13–15]. However, recent studies show a tendency towards more obese pediatric patients in the last couple of years, making this argument more valid in the future for the pediatric population [29–31]. Another patient group specifically benefiting from laparoscopic placement are patients with previous abdominal surgeries and possible adhesions. Laparoscopic placement has the advantage of directly visualising the tip of the VPS when placing it intraperitoneally, which was shown to have a benefit in patients with several previous abdominal surgeries and adhesions [14]. One classic subgroup of patients who fulfil these risk factors are premature new-borns, who have suffered from severe necrotizing enterocolitis (NEC). In this subgroup of patients accessing the abdomen can be challenging and they were shown to have a higher rate of shunt failure than premature new-borns without NEC [32]. No comparative study between laparoscopic and open technique exists in this subgroup. However, in the authors experience, in these children the abdomen is often accessed with assistance of the general surgeons regardless of other institutional standards. From the studies included in this meta-analysis, Hanna et al. excluded patients with previous abdominal surgery, while in the study by Fahy et al., both groups had a similar rate (3-5%) of patients who underwent previous abdominal surgery [13, 14]. Mulvaney et al. did not report if the patients included their study underwent previous abdominal surgery [15]. The study by Bani and Hassler, included in the qualitative systematic review, had the highest rate of previous abdominal surgery with 49%, while Soleman et al. reported a rate of 44% and Heye et al. a rate of 35% of patients with previous abdominal surgeries [19, 22, 24]. This difference could be due to different patient selection and could possibly introduce a selection bias.

Other studies in the literature described the use of the laparoscopic technique as standard for shunt revision surgeries in children but not for primary shunt insertions, while in some studies laparoscopic insertion resulted in fewer subsequent distal revision surgeries in children [33–35] 

The infection rate reported by the included studies remained under 10% without any significant difference between the laparoscopic and open group [13, 14, 24, 36, 37]. Whether laparoscopic VPS placement has an influence on the infection rate has been controversially discussed in the literature [11, 19, 25, 38, 39]. Further well-designed trials will need to investigate a possible lower infections rate with laparoscopy, since even a small difference, could influence the outcome significantly, as the burden of a VPS infection is tremendous for affected children. One concern with laparoscopic VPS placement, which is often performed by general surgeons, is that a higher number of surgeons and surgical equipment is required in the operating room, which was hypothesized to increase infection rates [40]. However, there are also reports of neurosurgeons performing laparoscopic VPS placement, which does not increase the number of the surgical team members present during surgery [21]. Hanna et al., Mulvaney et al. and Soleman et al. , explicitly stated that a pediatric surgeon performed laparoscopy, while Fahy et al. did not specify who performed the laparoscopy [13, 15, 24, 35]. In our experience, the absolute number of surgeons does not increase the infection rate, and on the contrary, two surgical teams working in parallel can shorten the procedure time, possibly reducing the risk of infections. Other potential factors influencing VPS infection rates is previous shunt surgery, the surgeons’ experience, duration of surgery, and type of shunt catheter [19, 41–43]. A recent randomized-controlled trial showed that antibiotic impregnated catheters have a significantly lower rate of infection, which could explain the high variability of the reported infection rates in the literature, especially from older studies during which such catheters have not been available yet [43]. The studies included in this meta-analysis did not mention whether an impregnated catheter was used [13–15]. Other factors such as the number of surgeons, gloving and handling of the shunt can have an impact on the infection rate [44]. In our practice, we implemented a no-touch technique for shunts and consequently double glove throughout the procedure, which was shown to reduce infections by other studies as well [44, 45]. None of the included studies mentioned the gloving process or other implemented techniques to reduce the infection rate specifically. Further, laparoscopic insertion using a periumbilical incision for the camera trocar and a small stab incision for a peel-away sheath to introduce the distal catheter under vision is a straightforward technique with an excellent cosmetic outcome [25, 46]. Another technique for VPS insertion in children is the blind trocar methods, where a small peel-away trocar is blindly inserted into the peritoneum. We have not included this technique in our review, since technically it is not similar to laparoscopic surgery, especially due to the fact that the insertion of the trocar and the catheter into the peritoneum is done blindly. In addition, only few studies are available describing the trocar insertion method [25, 47].

### Duration of surgery

Overall pooled surgical time was significantly shorter in the laparoscopic group. The study by Fahy et al. showed longer duration of surgery in the laparoscopic group, which could be either due to selection bias of more complex cases operated laparoscopically, or the need of two different surgical teams [14]. In the study by Fahy et al., Mulvaney et al., and Soleman et al. laparoscopic VPS placements were performed together with pediatric surgeons, while Hanna et al. did not specify who performed laparoscopy, which could also influence the difference in the observed surgery duration [13–15, 24]. In our experience, VPS surgery is done in parallel with the pediatric surgeons, which works logistically well and does not prolong the duration of surgery [24, 25, 45].

### Limitations

The present systematic review and meta-analysis has several limitations. First, we only searched two databases (PubMed and Embase) and only included articles in English, which could have led to omitting data published elsewhere or in a foreign language. Second, only few studies were available, which lead to heterogenous data included in this study, which could possibly bias the results of the pooled outcome analysis. Of these studies some were not comparative reports between the two methods but merely described their experience with laparoscopy, hence we only included three studies in the quantitative meta-analysis, which makes the analysis prone for statistical bias. Additionally, the studies included were all retrospective cohort studies, inherent to all limitations of such studies, which could influence the validity of our results. The follow-up interval varied among the different studies, which could influence their reported outcome. Finally, due to the possibility of unpublished negative studies, this analysis is inherent of a publication bias. The present study, however, includes a Iarge cohort of pediatric patients undergoing laparoscopic distal shunt insertion and is to our knowledge the first meta analyse analyzing the outcome of this technique.

## Conclusion

Laparoscopic VPS insertion is safe in children with similar distal revision and infection rates. Operation time was significantly shorter in the laparoscopic group. Based on the existing literature no firm conclusions can be drawn on the advantageous method for distal VPS placement, therefore prospective studies on the matter are needed.

## Data Availability

The data can be requested upon reasonable explanation from the corresponding author.
